# Compression Deformation Prediction of Chiral Metamaterials: A Compression–Shear Coupling Model

**DOI:** 10.3390/ma15155180

**Published:** 2022-07-26

**Authors:** Xin Zhou, Xi Liang, Zeliang Liu, Chenglin Tao, Huijian Li

**Affiliations:** 1 School of Civil Engineering and Mechanics, Yanshan University, Qinhuangdao 066004, China; lxzhouxin@126.com (X.Z.); taochenglin@stumail.ysu.edu.cn (C.T.); ysulhj@ysu.edu.cn (H.L.); 2 Hebei Key Laboratory of Mechanical Reliability for Heavy Equipments and Large Structures, Yanshan University, Qinhuangdao 066004, China

**Keywords:** chiral metamaterials, compression–shear coupling, ligament beam, elliptic integrals, large deformation

## Abstract

A category of metamaterials consisting of chiral cytosolic elements assembled periodically, in which the introduction of a rotatable annular structure gives metamaterials the ability to deform in compression–shear, has been a focus of research in recent years. In this paper, a compression–shear coupling model is developed to predict the compressive deformation behaviour of chiral metamaterials. This behaviour will be analysed by coupling the rotation of the annular node and the bending characteristics of ligament beam, which are obtained as a function of the length of ligament beam and the angle of rotation at the end of the beam. The shape function of the ligament beam under large deformation is obtained based on the elliptic integral theory; the function characterises the potential relationship between key parameters such as displacement and rotation angle at any point on the ligament beam. By simulating the deformation of cells under uniaxial compression, the reasonableness of the large deformation model of the ligament beam is verified. On this basis, a chiral cell-compression mechanical model considering the ductile deformation of the annular node is established. The compression–shear deformation of two-dimensional planar chiral metamaterials and three-dimensional cylindrical-shell chiral metamaterials was predicted; the offset displacements and torsion angles agreed with the experimental and finite element simulation results with an error of less than 10%. The developed compression–shear coupling model provides a theoretical basis for the design of chiral metamaterials, which meet the need for the precise control of shapes and properties.

## 1. Introduction

Metamaterials focus on the design of microstructure for different functions; for example, Lakes designed porous structures in two dimensions and analysed the negative Poisson’s ratio properties and deformation behaviour for these structures [1]. Subsequently, two-dimensional microstructures of chiral structure [2,3,4], star structure [5], and concave honeycomb structure [6,7] were developed, and the theoretical and experimental analyses of three-dimensional chiral metamaterials were carried out [8,9].

Chiral metamaterials are formed by tangentially connected annulars and elastic ligaments with periodic distributions to form a honeycomb topology, and to study their unique properties, the chiral single-cell [10], the design of anti-chiral metamaterials [11], multi-gradient chiral metamaterials [12], and the topological optimization design of chiral structures [13] have received extensive attention. The basic goal of topology optimisation is to obtain the optimal metamaterial properties for a specified volume. Many topology optimisation algorithms are shown for the design of metamaterials based on different strategies, such as the following: structural homogenisation theory [14], level-set functions [15,16], etc. Meanwhile, chiral metamaterials have been applied to deformed airfoils [17,18] and automotive manufacturing [19], etc. Deriving a non-linear mechanical model of the structure, analysing the relationship between non-linear stress and strain [20] and calculating the Poisson’s ratio of the structure under non-linear deformation [21] can help the structural design of metamaterials under different loads. Two-dimensional chiral metamaterials under uniaxial compression can exhibit large twists [22]. Non-linear rotatable mesh structures with high tensile properties were obtained by replacing the ligament straight beam with curved beams under uniaxial stretching conditions [23], achieving higher ductility and deformation properties of metamaterials. 

The current research objects mainly focus on mesh structures after cell combination and analyse its overall characteristics. For example, a prior study has examined the relationship between elastic modulus within a surface and Poisson’s ratio with geometrical parameters of structure in chiral metamaterials [24], the analysis of multi-chiral properties of the elastic modulus [25], and the derivation and evolution of Poisson’s ratio [26]. These conclusions mainly express the combinatorial properties composed of many cells and lack an effective mechanical behaviour description of a single-cell element. From a mechanistic analysis of the deformation of chiral structures, the rotation of single-cell annulus nodes and the bending of ligament beams are the main reasons for the large deformation of the structure [27].

The derivation of the chiral structure formulation is mostly based on the following assumptions: Annular nodes are completely rigid and deformation of annular nodes is not considered; forces perpendicular to the direction of applied load are neglected; internal forces depend on the observed deformation of the structure; based on the assumption of small deformation, only the elastic bending deformation of ligaments is considered and plastic deformation is neglected, and therefore the rotation of annular nodes is neglected [27]. Considering the to the above assumptions, the algorithm of numerical homogenisation is used to obtain the equivalent elastic moduli of metamaterials [28,29], the elastic deformation of a chiral-cell ligament beams is solved by using energy method [27], and the equivalent elastic moduli of irregular meshed metamaterials are calculated using a bottom-up analytical framework [30]. The application of chiral metamaterials usually results in large deflections, even if the metamaterials themselves are linearly elastic, leading to non-linear deformations of the ligament beam. Therefore, all these solutions ignore large deformations of the structure and will lead to large errors in predicting the deformation behaviours of metamaterials. The micropolar continuum model can overcome the above problems and derive a continuous function of chiral structural ligament beams and cell radii [31], which is more complicated. When considering the symmetry of chiral structure, special boundary conditions must be imposed on the cell.

In this paper, mesh structures made with three-dimensional printing (additive manufacturing) technology, a compression–shear coupling model, which considers the rotation of annular nodes, is established based on the design of tetrachiral cells to analyse the large deformation behaviours of ligament beam. The validity of the model is verified by comparison with finite-element simulations and experiments. Based on this model, the influence of geometric parameters on the deformation of meshes structure is investigated, and the deformation mechanism of meshes structure in the compression-shear process is characterised. The establishment of a compression–shear coupling model will provide a theoretical basis for the prediction and on-demand design of large deformations in chiral metamaterials.

## 2. A Mechanical Model for Large Deformations of Chiral Metamaterials under Uniaxial Compression

### 2.1. Parametric Analysis of Chiral Metamaterials

**Figure 1:**
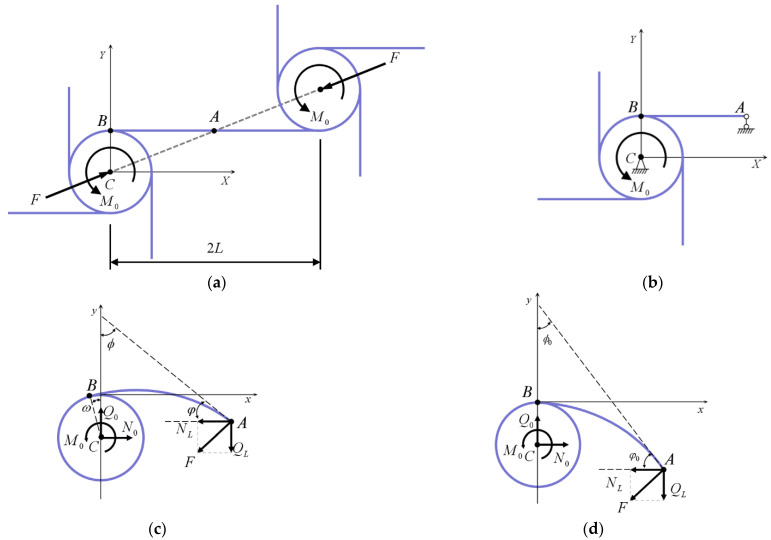
A schematic diagram of the deformation of ligament beams in chiral metamaterials (Point ‘A’ is the midpoint of the ligament beam connecting the twin cells, point ‘B’ is the connection point between the ligament beam and the annular node, and point ‘C’ is the center of the annular node). (**a**) A schematic diagram which shows the double cells subjected to axial force and bending movement; (**b**) A schematic diagram which shows the structure of the left half before deformation; (**c**) A schematic diagram which shows the mechanical model of ligament beam deformation after rotation of the annular node by ω; (**d**) A schematic diagram which shows the deformation of ligament beam without considering the rotations of annular nodes.

Under uniaxial compressions, the deformation characteristics of tetrachiral metamaterials may be expressed as two adjacent annular nodes centered by axial forces F and a pair of moments $M_{0}$, as shown in Figure 1a. Due to the anti-symmetry of the geometry, only half of the building-block structure must be analysed to obtain the undeformed schematic diagram of a single cell, as shown in Figure 1b. In this paper, only the case of equal length of each ligament beam is considered, and its length value is L. As shown in Figure 1c, the annular node is rotated around point ‘C’ where the angle of rotation is ω, the angle of rotation at any point on the ligament beam is ϕ, the angle between the tangent line of that point, and the y-axis is ϕ, $\phi \in \left[0,\pi /2\right]$. Figure 1d shows the deformation of the ligament beam without considerations of annular node rotation.

From Figure 1c, it can be seen that node ‘A’, under the action of an external force, can be decomposed into a component force $N_{L}$ in the horizontal projection direction and a component force $Q_{L}$ in the vertical projection direction along the coordinate system; where $N_{L}=nQ_{L}$, $F=\sqrt{1+n^{2}}Q_{L}$, n is the constant which represents the relationship between $N_{L}$ and $Q_{L}$. Without considering the displacement caused by the rotation of an annular node, the finite deformation of a chiral cell ligament beam can be reduced to a rod with one endpoint-fixed node and another endpoint forced, so that the beam bends in the plane with a large deflection. In such a rod segment, the angle between the tangent line and the y-axis is $\phi_{0}$, $\phi_{0}\in \left[0,\pi /2\right]$, as shown in Figure 1d:

(1) $$\left\{\begin{array}{c} dx/dl=\sin\phi_{0}\\ dy/dl=\cos\phi_{0} \end{array}\right.$$

where $\left(x,y\right)$ are the coordinates of the points on a ligament beam. The rotation angle of the chiral metamaterials contains two components: the angle change φ caused by the bending of the rod, and the angle change ω caused by the rotation of the annular node. This may be observed from the figure: $\phi_{0}=\frac{\pi }{2}-\varphi_{0}$, $\varphi_{0}=\varphi +\omega$, $\varphi =\frac{\pi }{2}+\phi$.

### 2.2. Equilibrium Equations for the Ligament Beam

**Figure 2:**
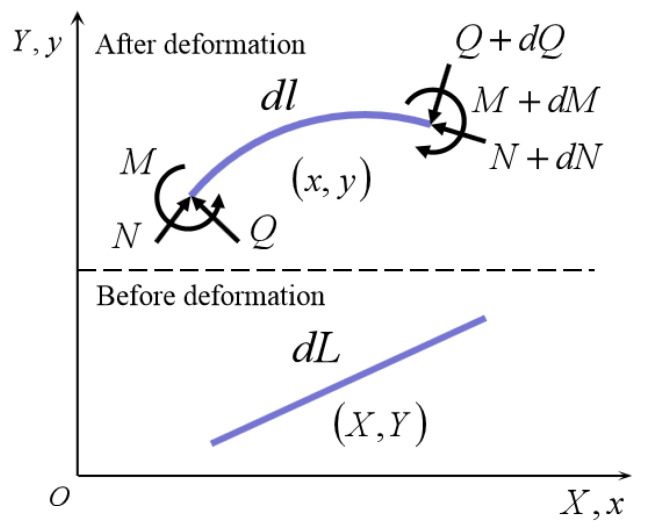
Deformation and internal forces in the ligament beam.

The micro-element dl on the deformed ligament beam is taken for force analysis, as shown in Figure 2. At the proximal face of dl, it is subjected to the action of M, N, and Q, where M is the moment acting on the micro-element synthesized by stress, and the moment acting on the distal section is M+dM. At equilibrium, the total moment acting on the micro-element must be equal to zero, as dM+dF×dl=0, where dl is the micro-element vector of the ligament beam.

Define r as the vector from a given point to any point on the ligament beam, where dl/dl=dr/dl, t is the unit tangent vector of the ligament beam, t=dl/dl. The moment of the ligament beam under bending action is $M=EIt\times \frac{dt}{dl}=EI\frac{dr}{dl}\times \frac{d^{2}r}{dl^{2}}$, and the equilibrium equation for the ligament beam bending is the following.

(2) $$EI\frac{dr}{dl}\times \frac{d^{3}r}{dl^{3}}=F\times \frac{dr}{dl}$$

### 2.3. Elliptic Integral Theory for Solving Large Deformations of Ligament Beam

In the deformation of chiral metamaterials under uniaxial compression, the two annular nodes are assumed to be rigid annulars which rotate around their centres and do not undergo any deformation. In order to convert the ligament beam-bending equilibrium equation as a function of ϕ, by expanding Equation (2) by the vector product, the vector product terms are as follows.

(3) $$\begin{array}{c} \frac{dr}{dl}=\sin\phi \cdot i+\cos\phi \cdot j\\ \frac{d^{2}r}{dl^{2}}=\cos\phi \frac{d\phi }{dl}\cdot i-\sin\phi \frac{d\phi }{dl}\cdot j\\ \frac{d^{3}r}{dl^{3}}=\left[-\sin\phi {\left(\frac{d\phi }{dl}\right)}^{2}+\cos\phi \frac{d^{2}\phi }{dl^{2}}\right]\cdot i+\left[-\cos\phi {\left(\frac{d\phi }{dl}\right)}^{2}-\sin\phi \frac{d^{2}\phi }{dl^{2}}\right]\cdot j \end{array}$$

Due to the complexity of the equation solution, this section solves the large deformation equation of the ligament beam based on the elliptic integral theory. After expanding the vector product of Equation (2) and taking one integral, define the relationship l with ϕ as follows:

(4) $$l=\pm \sqrt{\frac{EI}{2}}\int \frac{d\phi }{\sqrt{C_{1}-F\cos\phi }},$$

by solving the incomplete elliptic integral of first kind. Based on Equation (1), the coordinates for any point on ligament beam is obtained as follows.

(5) $$\left\{\begin{array}{l} x=\pm \frac{1}{F}\sqrt{2EI\left(C_{1}-F\cos\phi \right)}\\ y=\pm \sqrt{\frac{EI}{2}}\int \frac{\cos\phi }{\sqrt{C_{1}-F\cos}\phi }d\phi \end{array}\right.$$

As shown in Figure 1c, the fixed end of ligament beam (l=0) is defined as follows: ϕ=ω, free end (l=L): $\phi^{\prime }=0$, denote $\phi_{L}$ as the angle between the tangent to point ‘A’ at the free end of the ligament beam and the y-axis. Taking the above boundary conditions into Equation (4), the constant term under vertical force $Q_{L}$ is obtained as $C_{1}=Q_{L}\cos\phi_{Q_{L}}$, where $\phi_{Q_{L}}$ is recorded as $\phi_{L}$ solved by vertical force $Q_{L}$. Similarly, the constant term for the action of horizontal force $N_{L}$ can be solved as $N_{L}\cos\phi_{N_{L}}$. This produces an equation about $\phi_{L}$ as follows.

(6) $$\begin{array}{l} L=\sqrt{\frac{EI}{2Q_{L}}}\int_{\phi_{Q_{L}}}^{\frac{\pi }{2}-\omega }\frac{d\phi }{\sqrt{\cos\phi_{Q_{L}}-\cos\phi }}\\ L=\sqrt{\frac{EI}{2N_{L}}}\int_{\omega }^{\phi_{N_{L}}}\frac{d\phi }{\sqrt{\cos\phi -\cos\phi_{N_{L}}}} \end{array}$$

Therefore, when the end of the ligament beam is subjected to horizontal force $N_{L}$ and vertical force $Q_{L}$ in a chiral cell, the shape function of the large deformation of ligament beam may be solved as follows.

(7) $$\left\{\begin{array}{l} x\\ y \end{array}\right\}=\left\{\begin{array}{l} \sqrt{\frac{EI}{2N_{L}}}\int_{\omega }^{\phi_{L}}\frac{\cos\phi }{\sqrt{\cos\phi -\cos\phi_{L}}}d\phi +\sqrt{\frac{2EI}{Q_{L}}}\left(\sqrt{\cos\phi_{L}}-\sqrt{\cos\phi_{L}-\cos\phi }\right)\\ \sqrt{\frac{2EI}{N_{L}}}\left(\sqrt{1-\cos\phi_{L}}-\sqrt{\cos\phi -\cos\phi_{L}}\right)+\sqrt{\frac{EI}{2Q_{L}}}\int_{\phi_{L}}^{\frac{\pi }{2}-\omega }\frac{\cos\phi }{\sqrt{\cos\phi_{L}-\cos\phi }}d\phi \end{array}\right\}$$

As an example of uniaxial compression, the effective stress and effective strain are defined as follows [32]:

(8) $$\sigma_{\mathrm{eff}}=F/S,\;\varepsilon_{\mathrm{eff}}=1-L_{A}/L,$$

where $L_{A}=\sqrt{x_{A}^{2}+y_{A}^{2}}$, and S is the cross-sectional area of the ligament beam.

### 2.4. Small Deflection Deformation of the Ligament Beam

When the rotation of unit chiral cell annular node is small (that is, when a small deflection deformation of the ligament beam exists $\phi_{L}<<1$), based on the elliptic integral theory, the deformation of the free end of ligament beam can be approximated as follows.

(9) $$L\approx L_{A}=\sqrt{\frac{IE}{f}}\int_{0}^{\phi_{L}}\frac{d\phi }{\sqrt{\phi_{L}^{2}-\phi^{2}}}=\frac{\pi }{2}\sqrt{\frac{IE}{f}}$$

Thus, the critical force for the loss of linear form stability of the ligament beam is determined as follows.

(10) $$f\geq \pi^{2}IE/\left(4L^{2}\right),$$

This value also determines the limit value as a small deformation of the ligament beam.

### 2.5. Analysis of Cell Deformation under Uniaxial Compression

To verify the validity of the model, we used the commercial software ABAQUS (SIMULIA, Providence, RI, USA) for nonlinear finite element simulation. The beam cell (B22 element in ABAQUS) with refined meshes is adopted to ensure the accuracy of the calculation, the cross-sectional dimensions of the beam cells are 5 mm × 5 mm, and the radii of the annular nodes are all 20 mm.

The material’s elastic modulus is 1404.70 MPa, Poisson’s ratio is 0.3, and a 4 × 6 combination of cell is used. Based on Equation (6), the variation of $\phi_{L}$ with a load for different ligament lengths under uniaxial compression is shown in the figure below.

**Figure 3:**
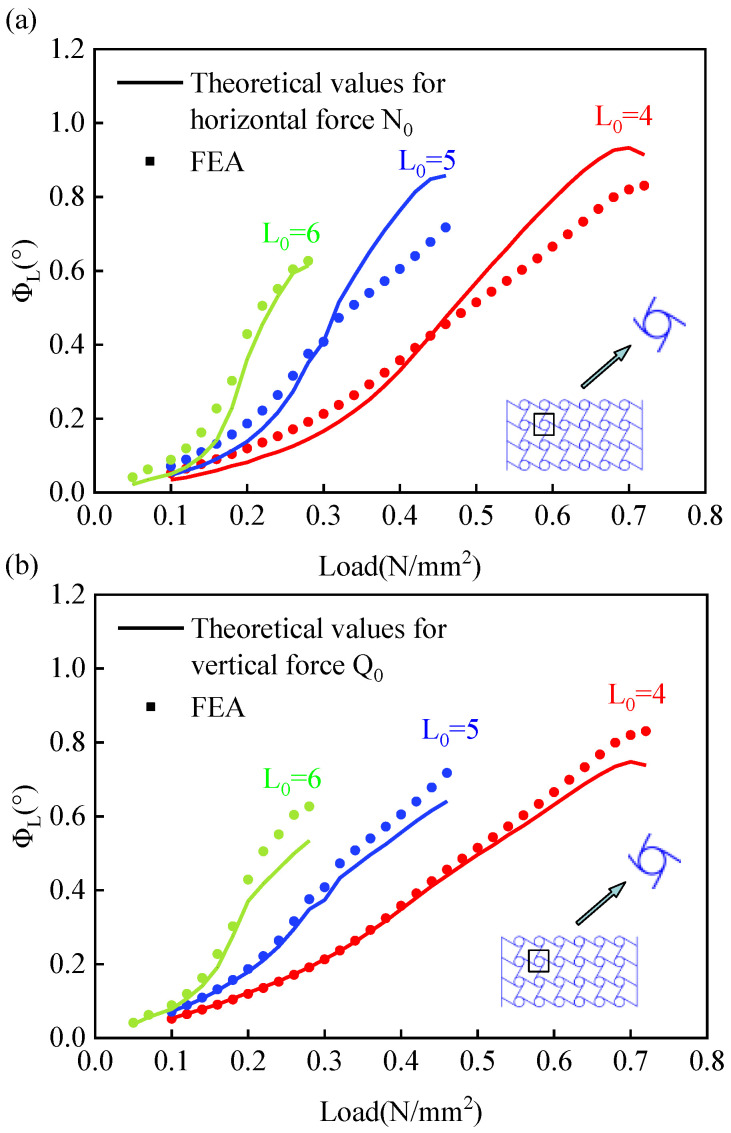
Curves of the rotation angle and load at node ‘A’ for different ligament beam lengths. (**a**) Rotation angle-load relationship curve under the action of horizontal force; (**b**) Rotation angle-load relationship curve under the action of verticalforce.

From the figures, it may be observed that the turning angle increases with increasing load values for the same ligament length. Under the same load, the rate of increase in node-turning angle is higher with increases in the ligament’s beam length. The ligament beam angles showed a “J” shape with the increased load, and the larger the ligament beam, the greater the slope. Comparing this finding with Figure 3a,b, it can be found that the theoretical values agree well with finite element simulation results, as the vertical forces tend to cause the ligament beam to lose its straight shape. A more accurate theoretical solution from the vertical force may be obtained during numerical iterations.

**Figure 4:**
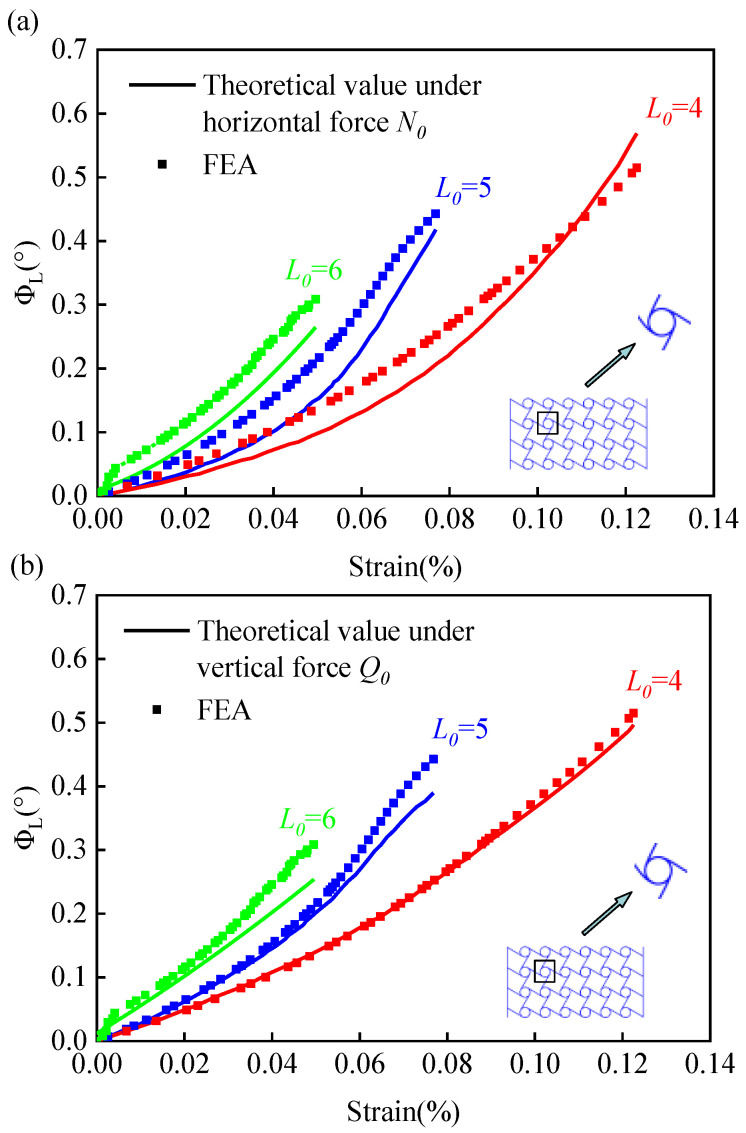
Curves of the rotation angle and strain at node ‘A’ for different ligament lengths. (**a**) Rotation angle-strain relationship curve under horizontal force; (**b**) Rotation angle-strain relationship curve under vertical force.

The relationship between rotation angle and strain for different directional forces is shown in Figure 4. It can be observed that, under the same rotation angle, the strain value decreases as the length of the ligament beam increases. Since ϕ contains the rotation angle of ligament beam end point ‘A’ and the rotation angle of annular node ‘B’, it can be observed in the figure that the larger the ligament beam, the less likely it will excite the rotation of the annular node under the condition of constant strain. Both Figure 4a,b show that the rotation angle increases with increasing strain at smaller strains; when the strain continues to increase, the rotation angle proceeds through a slow transition phase and enters a rapid increasing phase. In conclusion, the bending angle of the ligament beam calculated on the basis of elliptic integral theory agrees well with the results of finite element analysis.

**Figure 5:**
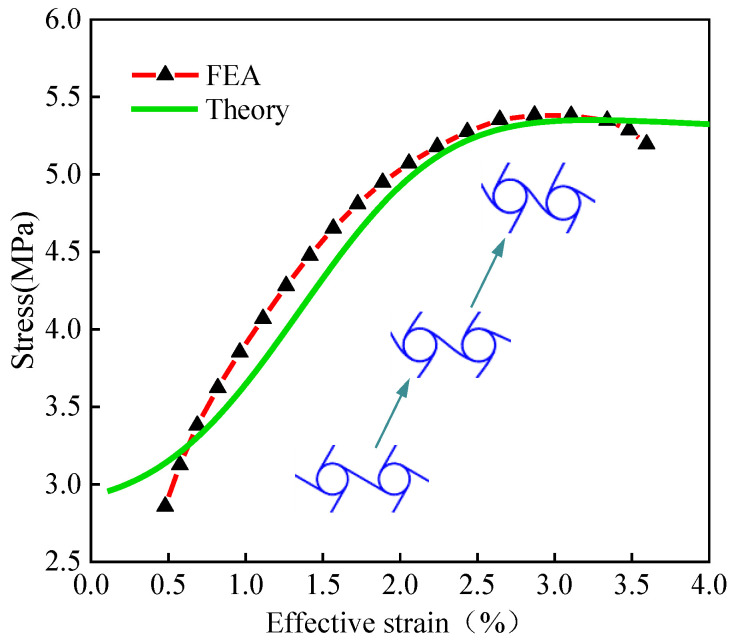
Effective strain-stress relationship curve for chiral metamaterials. (The length of the chiral ligament beam is 4 cm).

Taking the ligament beam length of 4 cm as an example, the effective strain of chiral metamaterials is calculated by Equation (8), and the effective strain–stress relationship is obtained, as shown in Figure 5.

It is shown in Figure 5 that the slope of the curve is consistent based on the theoretical value of the model’s solution and the finite element simulation result. The platform stress values of chiral metamaterials compressed to large deformations is consistent, and the yield strength of the mesh structure is much lower than the yield strength of the material itself (in this paper, the yield strength of the material is 100 MPa). In the compression process, the theoretical results are in good agreement with the finite element analysis results.

**Figure 6:**
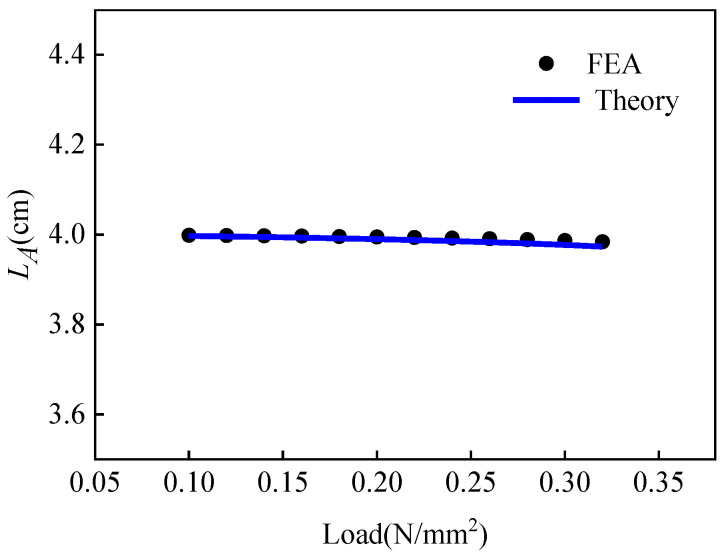
Curves of beam end deformation and load under small deformation (The length of the chiral ligament beam is 40 mm).

Based on the theoretical values of ligament beam deformations under small deflection deformation calculated by Equation (9), the finite element simulation results are positive, as shown in Figure 6. It can be seen from the figure that the curves are in good agreement, and the model has certain accuracy and applicability.

## 3. Ductile Deformation of Annular Nodes

### 3.1. Radial and Tangential Deformation under Annular Node Ductile Deformation

**Figure 7:**
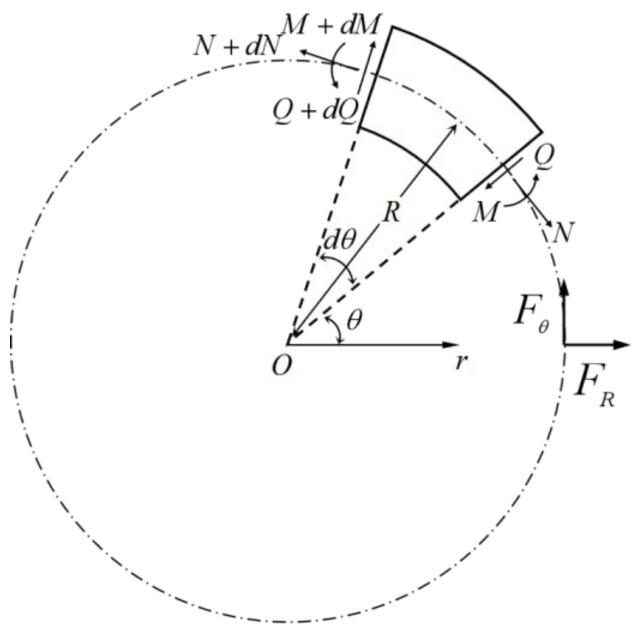
Force analysis of a micro-segment in the annular node.

With the compression of the tetrachiral cell, the ligament beam will undergo significant deformation, in addition to the large deformation of the annular nodes of the cell. Microsegments are selected at the angle of θ, R is the radius of the annulus, and tangential force $F_{\theta }$ and radial force $F_{R}$ are applied at the position θ=0, as shown in Figure 7.

In a microsegment, the relationship between radial internal force Q and radial external force $F_{R}$ is $d^{2}Q/d\theta^{2}=\left(Q/\pi \right)/\sin\theta -F_{R}$; hence, the general solution for the radial internal forces is described as follows.

(11) $$Q=C_{1}\cos\theta +C_{2}\cos\theta -\frac{1}{2\pi }F_{R}\theta \cos\theta$$

When the micro-segment is subjected to a radial force $F_{R}$, taking the moment at the midpoint of the micro-segment in Figure 7, based on the equilibrium equation for internal forces and moments with Equation (11), produces the following.

(12) $$N=-C_{1}\cos\theta +C_{2}\cos\theta +\frac{1}{2\pi }F_{R}\theta \sin\theta ,$$

(13) $$M=\left[C_{1}\sin\theta -C_{2}\cos\theta -\frac{1}{2\pi }F_{R}(\theta \sin\theta +\cos\theta )+C_{3}\right]R$$

From the above equation, introduce the relationship between bending moment, tangential internal force, and deformation [33].

(14) $$N=\frac{EA}{R}\left(\frac{\partial U_{\theta }}{\partial \theta }+U_{R}\right),\;M=\frac{EI}{R^{2}\left(1-\nu \right)}\left(\frac{\partial U_{\theta }}{\partial \theta }-\frac{\partial^{2}U_{R}}{\partial \theta^{2}}\right).$$

The higher-order non-simultaneous differential equations used to express the deformation of annular joints under the action of radial force are obtained as follows.

(15) $$\left\{\begin{array}{l} \frac{\partial^{5}U_{R}}{\partial \theta^{5}}+2\frac{\partial^{3}U_{R}}{\partial \theta^{3}}+\frac{\partial U_{R}}{\partial \theta }=-(\alpha +\beta )\sin\theta \\ \frac{\partial^{6}U_{\theta }}{\partial \theta^{5}}+2\frac{\partial^{4}U_{\theta }}{\partial \theta^{4}}+\frac{\partial^{2}U_{\theta }}{\partial \theta^{2}}=(\alpha +\beta )\sin\theta \end{array}\right.$$

Among them, we have $\alpha =\frac{RF_{R}}{\pi EA}$, $\beta =\frac{R^{3}F_{R}\left(1-\nu^{2}\right)}{\pi EI}$, and ν is Poisson’s ratio.

At the position of the polar axis, the annulus is not deformed in the radial or tangential direction. There is no tangential deformation at θ=π and it is mirrored symmetrically about the horizontal axis [34], as $V_{R\left(\theta =0\right)}=0$, $U_{\theta \left(\theta =0\right)}=0$, $U_{\theta \left(\theta =0\right)}=U_{\theta \left(2\pi -\theta \right)}$, $V_{\theta \left(\theta =0\right)}=V_{\theta \left(2\pi -\theta \right)}$. The analytical formulas of radial deformation and tangential deformation are obtained as follows.

(16) $$U_{R}=\frac{\alpha }{4}\left[\left(\theta -\pi \right)\sin\theta +\left(\theta \pi -\frac{\theta^{2}}{2}\right)\cos\theta \right]+\frac{\beta }{4}\left[\left(\theta -\pi \right)\sin\theta +\left(2+\theta \pi -\frac{\theta^{2}}{2}\right)\cos\theta -2\right],\;U_{\theta }=\frac{\alpha }{4}\sin\theta +\frac{\beta }{4}\left[2\pi -\theta +\left(\theta -2\pi \right)\cos\theta +\left(\frac{\theta^{2}}{2}-\theta \pi \right)\sin\theta \right].$$

To solve the radial displacement and tangential displacement under the action of tangential force $F_{\theta }$, a balance equation of force and moment under tangential force is constructed, and the relationship between bending moment, tangential internal force, and radial deformation is introduced through the balance equation of the force and moment. The higher-order inhomogeneous differential equations used to express the deformation of an annular node under tangential force are obtained.

(17) $$\left\{\begin{array}{l} \frac{\partial^{5}U_{R}}{\partial \theta^{5}}+2\frac{\partial^{3}U_{R}}{\partial \theta^{3}}+\frac{\partial U_{R}}{\partial \theta }=(\alpha +\beta )\cos\theta +\frac{\beta }{2}\\ \frac{\partial^{6}U_{\theta }}{\partial \theta^{5}}+2\frac{\partial^{4}U_{\theta }}{\partial \theta^{4}}+\frac{\partial^{2}U_{\theta }}{\partial \theta^{2}}=-(\alpha +\beta )\cos\theta -\frac{\beta }{2} \end{array}\right.$$

Among them, we have $\alpha =\frac{RF_{R}}{\pi EA}$, $\beta =\frac{R^{3}F_{R}\left(1-\nu^{2}\right)}{\pi EI}$, and ν is Poisson’s ratio. Then, the analytical equations for radial and tangential deformation are

(18) $$U_{R}=\frac{\alpha }{4}\left[\frac{5\pi^{2}}{8}+\pi \theta -\frac{\theta^{2}}{2}\right]\sin\theta +\frac{\beta }{2}\left[\left(\frac{\pi \theta }{2}+\frac{\pi }{2}-\frac{3\pi^{2}}{64}-\frac{\theta^{2}}{4}\right)\sin\theta +\left(\pi -\theta \right)\left(\cos\theta -1\right)\right],\;U_{\theta }=\frac{\alpha }{4}\left[\begin{array}{l} \left(\frac{5\pi^{2}}{8}-2-\frac{\theta^{2}}{2}+\theta \pi \right)\cos\theta \\ +\left(1-\theta \right)\pi \sin\theta \end{array}\right]+\frac{\beta }{4}\left[\begin{array}{l} \left(\theta \pi +\frac{\left(6-\theta^{2}\right)}{2}+\pi -\frac{15\pi^{2}}{32}\right)\cos\theta \\ +3\left(\theta -\pi \right)\sin\theta +\theta \left(2\pi -\theta \right)-3 \end{array}\right].$$

The analytical equations for the radial and tangential deformations of the annular node under the action of the bending moment are as follows [34].

(19) $$U_{R}=-\frac{MR^{2}}{EI}\left[\left(1-\frac{\theta }{\pi }\right)\frac{\left(1-\cos\theta \right)}{2}-\frac{3\sin\theta }{4\pi }\right],\;U_{\theta }=-\frac{MR^{2}}{EI}\left(\frac{\cos\theta }{\pi }+\frac{1}{2\pi }\right)$$

### 3.2. Compression-Shear Deformation of a Chiral Single Cell

**Figure 8:**
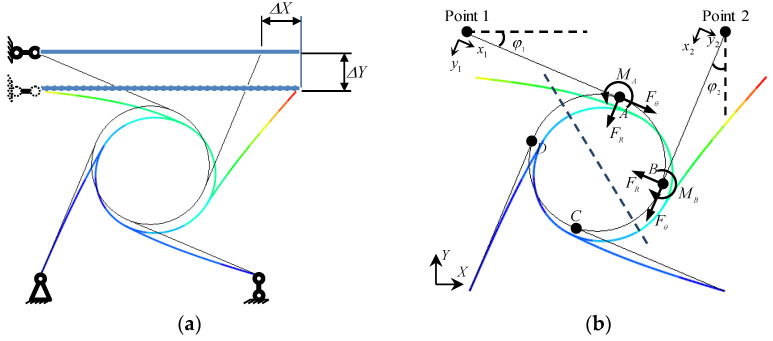
A schematic diagram of a tetrachiral cell. (**a**) Single-cell displacement and constraint; (**b**) A schematic diagram of the mechanics model for a chiral cell.

The chiral cell has a simple and variable structure (few parameters and easy to control) with guided deformation, and it exhibits properties of circular deformation and rotation under compression, as shown in Figure 8a. To describe the effect of ligament beam bending on the annular node, the mechanical model is shown in Figure 8b, based on the theoretical solution of Section 3.1. the deformation of the annular node in a tetrachiral cell is obtained as follows:

(20) $$U_{RRij}=\frac{\alpha }{4}\left[\left(\theta_{ij}-\pi \right)\sin\theta_{ij}+\left(\theta_{ij}\pi -\frac{\theta_{ij}{}^{2}}{2}\right)\cos\theta_{ij}\right]+\frac{\beta }{4}\left[\left(\theta_{ij}-\pi \right)\sin\theta_{ij}+\left(2+\theta_{ij}\pi -\frac{\theta_{ij}{}^{2}}{2}\right)\cos\theta_{ij}-2\right],\;U_{R\theta ij}=\frac{\alpha }{4}\sin\theta_{ij}+\frac{\beta }{4}\left[2\pi -\theta_{ij}+\left(\theta_{ij}-2\pi \right)\cos\theta_{ij}+\left(\frac{\theta_{ij}{}^{2}}{2}-\theta_{ij}\pi \right)\sin\theta_{ij}\right],\;U_{\theta Rij}=\frac{\alpha }{4}\left[\frac{5\pi^{2}}{8}+\pi \theta_{ij}-\frac{\theta_{ij}{}^{2}}{2}\right]\sin\theta_{ij}+\frac{\beta }{2}\left[\left(\frac{\pi \theta_{ij}}{2}+\frac{\pi }{2}-\frac{3\pi^{2}}{64}-\frac{\theta_{ij}{}^{2}}{4}\right)\sin\theta_{ij}+\left(\pi -\theta_{ij}\right)\left(\cos\theta_{ij}-1\right)\right],\;U_{\theta \theta ij}=\frac{\alpha }{4}\left[\begin{array}{l} \left(\frac{5\pi^{2}}{8}-2-\frac{\theta_{ij}{}^{2}}{2}+\theta \pi \right)\cos\theta_{ij}\\ +\left(1-\theta \right)\pi \sin\theta_{ij} \end{array}\right]+\frac{\beta }{4}\left[\begin{array}{l} \left(\theta_{ij}\pi +\frac{\left(6-\theta_{ij}{}^{2}\right)}{2}+\pi -\frac{15\pi^{2}}{32}\right)\cos\theta_{ij}\\ +3\left(\theta_{ij}-\pi \right)\sin\theta_{ij}+\theta_{ij}\left(2\pi -\theta_{ij}\right)-3 \end{array}\right]$$

where $U_{RRij}$ denotes the radial displacement of point j by the radial load at point i, $U_{\theta Rij}$ denotes the radial displacement of point j by the tangential load at point i, $U_{R\theta ij}$ denotes the tangential displacement of point j by the radial load at point i, and $U_{\theta \theta ij}$ denotes the tangential displacement of point j by the tangential load at point i.

Based on elliptical integration theory, $\left(x_{1},y_{1}\right)$ is the coordinate of the free end of the ‘1-A’ section of the cantilever beam in the local coordinate system, and $\left(x_{2},y_{2}\right)$ is the coordinate of the free end of the cantilever beam ‘2-B’ in the local coordinate system. The beam’s end displacements can be found for a tetrachiral cell, taking into account the ductile deformation of the annulus as follows.

(21) $$\Delta Y_{1}=\left[\begin{array}{l} y_{1}+\left(\begin{array}{l} U_{RRAA}+U_{\theta RAA}+\\ U_{RRBA}+U_{\theta RBA} \end{array}\right)\\ +\left(\begin{array}{l} U_{RRAD}+U_{\theta RAD}+\\ U_{RRBD}+U_{\theta RBD} \end{array}\right) \end{array}\right]\cos\varphi_{1}+\left[\begin{array}{l} \left(L-x_{1}\right)+\left(\begin{array}{l} U_{R\theta AA}+U_{\theta \theta AA}+\\ U_{R\theta BA}+U_{\theta \theta BA} \end{array}\right)\\ +\left(\begin{array}{l} U_{R\theta AC}+U_{\theta \theta AC}+\\ U_{R\theta BC}+U_{\theta \theta BC} \end{array}\right) \end{array}\right]\sin\varphi_{1},\;\Delta X_{1}=\left[\begin{array}{l} y_{1}+\left(\begin{array}{l} U_{RRAA}+U_{\theta RAA}+\\ U_{RRBA}+U_{\theta RBA} \end{array}\right)\\ +\left(\begin{array}{l} U_{RRAD}+U_{\theta RAD}+\\ U_{RRBD}+U_{\theta RBD} \end{array}\right) \end{array}\right]\sin\varphi_{1}-\left[\begin{array}{l} \left(L-x_{1}\right)+\left(\begin{array}{l} U_{R\theta AA}+U_{\theta \theta AA}+\\ U_{R\theta BA}+U_{\theta \theta BA} \end{array}\right)\\ +\left(\begin{array}{l} U_{R\theta AC}+U_{\theta \theta AC}+\\ U_{R\theta BC}+U_{\theta \theta BC} \end{array}\right) \end{array}\right]\cos\varphi_{1},\;\Delta Y_{2}=\left[\begin{array}{l} y_{2}+\left(\begin{array}{l} U_{RRAB}+U_{\theta RAB}+\\ U_{RRBB}+U_{\theta RBB} \end{array}\right)\\ +\left(\begin{array}{l} U_{RRAD}+U_{\theta RAD}+\\ U_{RRBD}+U_{\theta RBD} \end{array}\right) \end{array}\right]\sin\varphi_{2}+\left[\begin{array}{l} \left(L-x_{2}\right)+\left(\begin{array}{l} U_{R\theta BB}+U_{\theta \theta BB}+\\ U_{\theta \theta AB}+U_{R\theta AB} \end{array}\right)\\ +\left(\begin{array}{l} U_{R\theta BD}+U_{\theta \theta BD}+\\ U_{\theta \theta AD}+U_{R\theta AD} \end{array}\right) \end{array}\right]\cos\varphi_{2},\;\Delta X_{2}=\left[\begin{array}{l} y_{2}+\left(\begin{array}{l} U_{RRAB}+U_{\theta RAB}+\\ U_{RRBB}+U_{\theta RBB} \end{array}\right)\\ +\left(\begin{array}{l} U_{RRAD}+U_{\theta RAD}+\\ U_{RRBD}+U_{\theta RBD} \end{array}\right) \end{array}\right]\cos\varphi_{2}-\left[\begin{array}{l} \left(L-x_{2}\right)+\left(\begin{array}{l} U_{R\theta BB}+U_{\theta \theta BB}+\\ U_{\theta \theta AB}+U_{R\theta AB} \end{array}\right)\\ +\left(\begin{array}{l} U_{R\theta BD}+U_{\theta \theta BD}+\\ U_{\theta \theta AD}+U_{R\theta AD} \end{array}\right) \end{array}\right]\sin\varphi_{2}$$

## 4. Deformation Behaviour Analysis of Chiral Metamaterials under Uniaxial Compression

To investigate the controlled and versatile properties of chiral metamaterials, to achieve a change from passive deformation to controlled directional deformation, and to meet a range of specific deformation requirements, find the combined structure of two types of chiral cells and analyse their deformation properties.

### 4.1. Structural Design of Chiral Metamaterials

**Figure 9:**
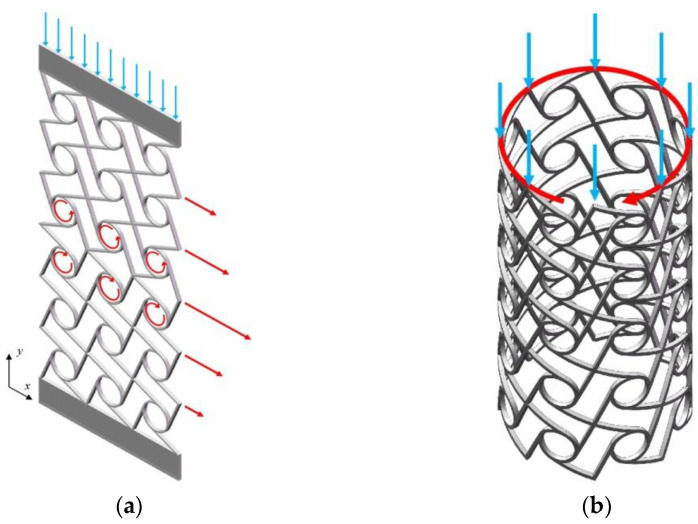
The structural design of chiral metamaterials. (**a**) Two-dimensional planar structure; (**b**) Three-dimensional cylindrical shell structure.

To achieve the controlled deformation of chiral metamaterials, the corner and displacement of key nodes are two main judging factors [35]. Based on a tetrachiral cell structure, these structures shown in Figure 9 do not undergo significant normal expansion under vertical pressure but have compression–shear characteristics.

### 4.2. Compression-Shear Coupling Deformation of a Two-Dimensional Planar Structure

**Figure 10:**
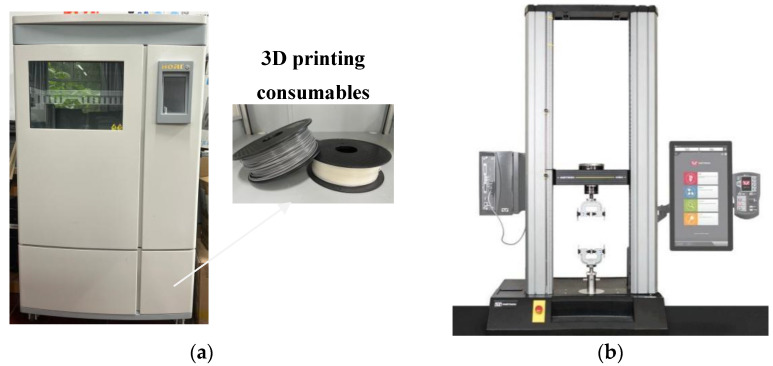
Experimental apparatus. (**a**) 3D printer; (**b**) Universal testing machine.

**Figure 11:**
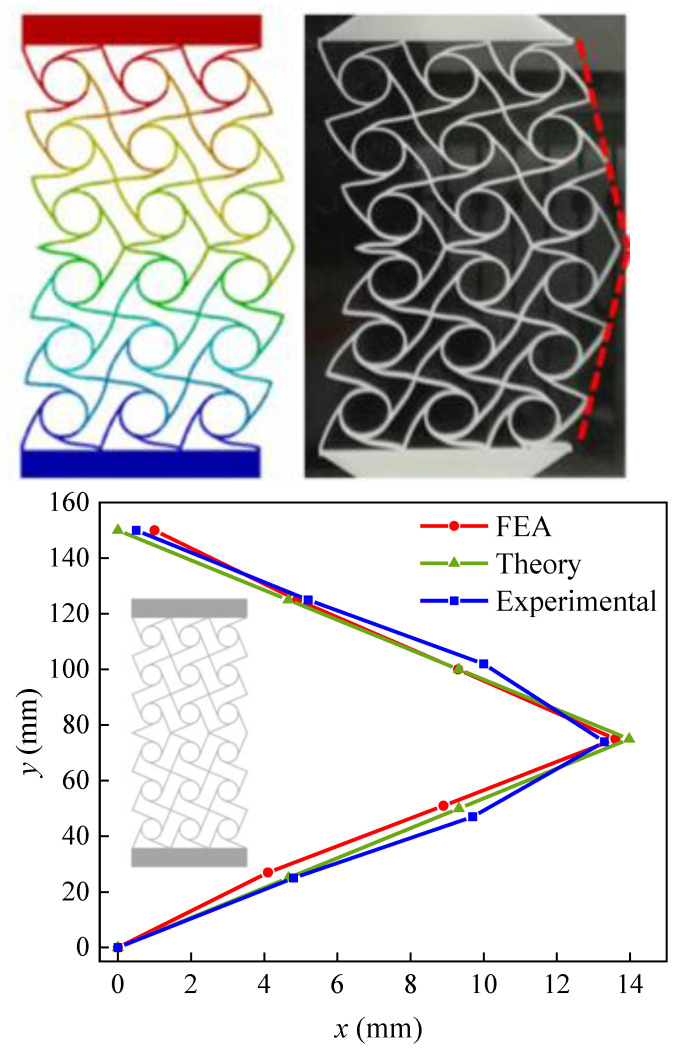
Two-dimensional planar compression–shear coupling deformation of chiral cells. (The red dotted line is the compression-shear deformation on one side of the structure).

This example relies on three-dimensional printing technology to place the cured sample in a universal testing machine for compression testing. The loading rate of the compression test is 5 mm/min. The instrument used in this experiment is shown in Figure 10. The two-dimensional planar structure is divided into six rows and three columns, with the lower three rows being left-handed chiral cells and the upper three rows being right-handed chiral cells, all with the same cell geometry. Each cell is subjected to the same load in the vertical direction, and deformation occurs relatively independently. Since the deformation is vector-additive [36], the sum of the deformation vectors of the lower three rows of cells results in a clockwise tilt of the right side of the profile as an oblique line. Due to the rigid connection of the two rows of different chiral cells at the middle symmetry axis, the deformation is influenced by the transferred bending moment, resulting in a deviation between the actual deformation and the target deformation, with an overall appearance of “>”, as shown in Figure 11. When the structure is compressed to the cell deformation as shown in Figure 11, the internal force at point ‘1’ of the unit cell at the bottom of the structure is 0.21 N, and the internal force at point ‘2’ is 0.145 N. Through the numerical iteration of the elliptic integral, the rotation angle of point ‘1’ is 15.76°, and the rotation angle of point ‘2’ is 17.77°. Based on the calculations of this model, the theoretical values of chiral single-cell deformations were obtained, as shown in Table 1. The table also contains the combined dimensional configuration and material information for a two-dimensional mesh structure. The final displacement in the compression direction (Y-direction) at beam end point ‘1’ of the single cell is 5.24 mm, the displacement in the compression direction (X-direction) at beam end point ‘1’ is 0.65 mm, the displacement in the compression direction (Y-direction) at beam end point ‘2’ is 4.92 mm, and the displacement in the compression direction (X-direction) at beam end point ‘2’ is 4.66 mm. Figure 11 shows the FEM simulation deformation results, the experimental deformation results, and the theoretical calculation results, which are in better agreement; the error is less than 10%.

### 4.3. Compression-Torsion Coupling Deformation of a Cylindrical Shell Structure

**Figure 12:**
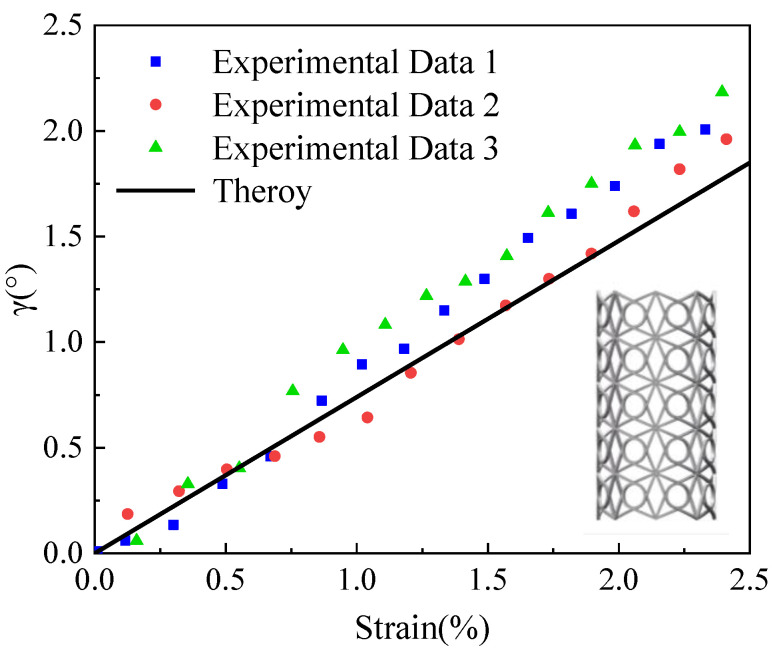
Three-dimensional cylindrical shell compression-torsion coupling deformations of chiral cells.

The torsion angle γ of this cylindrical shell structure can be derived from the lateral displacement of the planar chiral single cell. If the lateral displacement of the planar chiral single cell is approximated as the circumferential displacement of the chiral cylindrical shell structure, then $\gamma =\Delta u/R^{\prime }$, $R^{\prime }$ is the radius of the cylindrical shell structure. The experiment was carried out using a low-viscosity photosensitive resin based on three-dimensional printing technology, the cell dimensions were 15 mm × 15 mm, the elastic modulus of the material is 1785.60 MPa, the Poisson’s ratio is 0.4, the number of circumferential cells in the cylindrical shell is eight, and the number of axial cells is five. The curve plot of strain versus torsion angle is shown in Figure 12. It can be observed from the graph that the theoretical values of the obtained torsional rates and the experimental results [37] are in good agreement.

## 5. Conclusions

For chiral metamaterials, a non-linear theoretical model is established in this paper for predicting the deformation of structure and solving shape function for the large deformation of ligament beam. Based on the theoretical solutions of elliptic integral and finite element simulation, the relationship curves of rotation angle, strain, and load under different ligament lengths are obtained, which verifies that the model can effectively couple the two mechanical responses of ring node rotations and ligament beam bending. The theoretical values are in good agreement with the numerical simulations.

For the study of chiral cell deformation, it is assumed that the ring nodes are completely rigid; that is, the deformation of the annular nodes is not considered in this paper. Based on the established compression–shear coupling deformation model, large deformations of ligament beam, accounting for the ductile deformation of cell annular nodes coupled with the vector additivity of the deformation of cells under unidirectional loading, enable metamaterials to achieve specific deformations. Compression tests of two types of chiral-cell combined structures are based on three-dimensional printing technology. The results show that the theoretical model is able to fit the experimental results and can be used as a theoretical basis for achieving a controlled deformation mechanical response of chiral metamaterials, providing a systematic guide for solving large deformations of chiral structures.

## Figures and Tables

**Table 1:** Displacements of ligament beam endpoints.

Combined Configurations	Theoretical Solutions	Radial Displacement at Point ‘1’ (mm)	Tangential Displacement at Point ‘1’ (mm)	Radial Displacement at Point ‘2’ (mm)	Tangential Displacement at Point ‘2’ (mm)
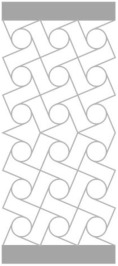	Elliptic integral solution	5.06	0.36	6.02	1.17
	Annular node ductile deformation	−0.14	1.08	0.13	1.55
	Material properties	The material properties are photosensitive resin materials, the cell size is 30 mm × 30 mm, the Elastic modulus is 1404.70 MPa, and the Poisson’s ratio is 0.3. The diameter of the annular node is 16 mm, and the width of the ligament beam is 1 mm.			

## Data Availability

Not applicable.
